# Genetic variants in *PTPRD* and risk of gestational diabetes mellitus

**DOI:** 10.18632/oncotarget.12599

**Published:** 2016-10-12

**Authors:** Ting Chen, Juan Xu, Guangquan Liu, Heng Liu, Minjian Chen, Yufeng Qin, Wei Wu, Yankai Xia, Chenbo Ji, Xirong Guo, Juan Wen, Xinru Wang

**Affiliations:** ^1^ State Key Laboratory of Reproductive Medicine, Institute of Toxicology, Nanjing Medical University, Nanjing 211166, China; ^2^ Key Laboratory of Modern Toxicology of Ministry of Education, School of Public Health, Nanjing Medical University, Nanjing 211166, China; ^3^ Nanjing Maternity and Child Health Care Institute, Nanjing Maternity and Child Health Care Hospital Affiliated to Nanjing Medical University, Nanjing 210004, China; ^4^ Department of Obstetrics and Gynecology, Nanjing Maternity and Child Health Care Hospital Affiliated to Nanjing Medical University, Nanjing 210004, China; ^5^ Epigenetics & Stem Cell Biology Laboratory, National Institute of Environmental Health Sciences, Research Triangle Park, NC 27709, USA; ^6^ Department of Children Health Care, Nanjing Maternity and Child Health Care Hospital Affiliated to Nanjing Medical University, Nanjing 210004, China

**Keywords:** PTPRD, polymorphism, gestational diabetes mellitus, susceptibility

## Abstract

Genome-wide association studies (GWASs) showed that two single nucleotide polymorphisms (SNPs) (rs17584499 and rs649891) in the protein tyrosine phosphatase receptor type D (*PTPRD*) were associated with type 2 diabetes (T2D). We sought to determine the influence of the *PTPRD* variants on the gestational diabetes mellitus (GDM) risk. In this research, two SNPs in *PTPRD* reported in T2D GWASs and six *PTPRD* expression-related SNPs were genotyped in 964 GDM cases and 1,021 controls using the Sequenom platform. Logistic regression analyses in additive models showed consistently significant associations of *PTPRD* rs10511544 A>C, rs10756026 T>A and rs10809070 C>G with a decreased risk of GDM [adjusted OR (95% CI) = 0.83 (0.72-0.97) for rs10511544; adjusted OR (95% CI) = 0.81 (0.70-0.94) for rs10756026; adjusted OR (95% CI) = 0.78 (0.65-0.92) for rs10809070]. Furthermore, the risk of GDM was significantly decreased with an increasing number of variant alleles of the three SNPs in a dose-dependent manner (*P*_trend_ = 0.008). Moreover, the haplotype containing variant alleles of the three SNPs were significantly associated with a decreased risk of GDM [adjusted OR (95% CI) = 0.77 (0.64-0.92), *P* = 0.005], when compared with the most frequent haplotype. However, there were no significant associations for the SNPs reported in the T2D GWASs. Altogether, these findings indicate that the variants of rs10511544, rs10756026 and rs10809070 in *PTPRD* may contribute to a decreased susceptibility to GDM. Further validation in different ethnic backgrounds and biological function analyses are needed.

## INTRODUCTION

Gestational diabetes mellitus (GDM) is defined as varying degrees of glucose intolerance with an onset or first recognition during pregnancy [1]. The incidence of GDM has steadily risen in the last few decades and affects 1%-14% of all pregnancies [2]. Both GDM patients and their children have an increased risk of developing type 2 diabetes (T2D) and metabolic syndrome later in life [3]. Human and animal studies suggested that T2D and GDM may have some common pathological changes, including insulin resistance, β-cell dysfunction and defects in insulin action [4], and may share predisposing factors. As there is evidence for a higher prevalence of T2D in mothers of GDM patients and the clustering of impaired glucose tolerance and T2D in families with GDM patients [5], GDM may have similar genetic background as T2D.

In the past few decades, genetic studies, including genome-wide association studies (GWASs) and candidate gene strategies, have identified many susceptibility genes involved in the increased risk of T2D and GDM [6, 7]. There has been progress in finding the genetic risk factors for GDM in relation to T2D. Some of the genetic variants that were proven to be genetic risk factors for T2D are also significantly associated with GDM [7, 8]. In 2010, Tsai et al. performed a two-stage GWAS (995 T2D patients and 894 controls for the first genome scan) and identified rs17584499 in the protein tyrosine phosphatase receptor type D (*PTPRD*) gene for T2D in a Han Chinese population [9]. Then, Below et al. performed another GWAS with 837 T2D cases and 436 normoglycaemic controls for a GWA scan, followed by a meta-analysis, and found such an association with another SNP, rs649891, in *PTPRD* in Mexican-Americans [10]. In a replication study, the *PTPRD* genetic variant was suggested to be associated with progression to diabetes in Han Chinese, most likely through increased insulin resistance [11]. Recently, Chen et al. found that the levels of PTPRD were significantly lower in T2D patients, and this protein was involved in the insulin signalling pathway [12]. However, few studies have investigated the associations of the genetic variants of *PTPRD* with the risk of GDM.

In a Korean population, some of the T2D-associated genetic variants discovered in GWASs were suggested to also be associated with GDM [7]. Therefore, in this study, we hypothesized that genetic variants in *PTPRD* might also contribute to the risk of GDM. To test the hypothesis, we selected 8 single nucleotide polymorphisms (SNPs) in *PTPRD* and performed a case-control study, including 964 GDM cases and 1,021 controls, to test the associations of the SNPs with the risk of GDM.

## RESULTS

The selected characteristics of the 964 GDM patients and 1,021 controls are shown in Table 1. As expected, there were similar distributions of age and pre-pregnancy body mass index (BMI) between the two groups (*P* = 0.094 and 0.685). However, there were more multiparaes, women with abnormal pregnancy histories and women with family histories of diabetes among the GDM cases than among the controls (*P* < 0.05 for all comparisons).

**Table 1:** Demographic and selected variables in GDM cases and controls

Variables	GDM cases (n = 964) N (%)	Controls (n = 1021) N (%)	*P*
Age, year (mean ± SD)	30.6 ± 3.7	30.3 ± 3.6	0.094
Pre-pregnancy BMI, kg/m^2^ (mean ± SD)	22.1 ± 2.9	22.0 ± 2.8	0.685
Parity			< 0.001
Nulliparae	827 (85.8)	953 (93.3)	
Multiparae	137 (14.2)	68 (6.7)	
Abnormal pregnancy history			< 0.001
No	847 (87.9)	981 (96.1)	
Yes	117 (12.1)	40 (3.9)	
Family history of diabetes			0.023
No	791 (82.1)	876 (85.8)	
Yes	173 (17.9)	145 (14.2)	

Abbreviation: GDM, gestational diabetes mellitus; SD, standard deviation; BMI, body mass index.

Logistic regression analyses in different genetic models were used to examine the associations between the 8 studied SNPs and GDM susceptibility. Among the controls, the genotype frequencies of the 8 SNPs were all in Hardy-Weinberg equilibrium (*P* > 0.05 for all SNPs). We did not validate the results of the *PTPRD* variations (rs17584499 and rs649891) reported in the published GWASs for T2D, but we observed that *PTPRD* rs10511544 A>C, rs10756026 T>A and rs10809070 C>G were significantly associated with a decreased risk of GDM using the additive model [adjusted OR (95% CI) = 0.83 (0.72-0.97) for rs10511544; adjusted OR (95% CI) = 0.81 (0.70-0.94) for rs10756026; adjusted OR (95% CI) = 0.78 (0.65-0.92) for rs10809070] (Table 2–3). Then, conditional logistic regression analyses were used to test the independence of the three significant SNPs. The effects of rs10511544 and rs10809070 on GDM occurrence were weakened after being conditioned on the other two SNPs. However, the effect of rs10756026 on GDM occurrence persisted after being conditioned on the other two SNPs [adjusted OR (95% CI) = 0.71 (0.51-0.99)].

**Table 2:** Genotyping results in GDM cases and controls

SNP	Base chang^a^	MAF^b^	Reported MAF^c^	*P* value		
				Dominant model	Recessive model	Additive model
rs17584499	C>T	0.090	0.087	0.331	0.076	0.190
rs649891	C>T	0.217	0.223	0.418	0.555	0.654
rs10511544	A>C	0.267	0.267	0.116	0.003	0.015
rs10756026	T>A	0.253	0.262	0.041	0.003	0.005
rs10809070	C>G	0.180	0.175	0.047	< 0.001	0.004
rs12345848	G>A	0.177	0.180	0.546	0.134	0.318
rs1323500	T>G	0.434	0.437	0.370	0.218	0.200
rs628731	A>G	0.250	0.257	0.243	0.013	0.058

a Notes: Major > minor allele;

b MAF in 1021 controls;

c Reported MAF in Han Chinese from 1,000 genomes. Abbreviations: GDM, gestational diabetes mellitus; SNP, single nucleotide polymorphism; MAF, minor allele frequency.

**Table 3:** Association between 3 significant SNPs and GDM susceptibility

Genotype	GDM cases (n = 964) N (%)	Controls (n = 1021) N (%)	OR (95%CI)	*P*	OR (95%CI)^a^	*P*^a^
rs10511544						
AA	562 (58.7)	559 (54.8)	1.00		1.00	
CA	348 (36.3)	378 (37.0)	0.92 (0.76-1.10)	0.356	0.93 (0.77-1.13)	0.472
CC	48 (5.0)	84 (8.2)	0.57 (0.39-0.83)	0.003	0.56 (0.38-0.82)	0.003
Additive			0.83 (0.72-0.96)	0.011	0.83 (0.72-0.97)	0.015
rs10756026						
TT	591 (61.4)	573 (56.6)	1.00		1.00	
AT	332 (34.5)	366 (36.2)	0.88 (0.73-1.06)	0.180	0.89 (0.73-1.07)	0.217
AA	40 (4.2)	73 (7.2)	0.53 (0.36-0.79)	0.002	0.53 (0.35-0.80)	0.002
Additive			0.81 (0.70-0.93)	0.004	0.81 (0.70-0.94)	0.005
rs10809070						
CC	691 (72.9)	700 (68.6)	1.00		1.00	
GC	241 (25.4)	273 (26.8)	0.89 (0.73-1.10)	0.280	0.90 (0.73-1.11)	0.313
GG	16 (1.7)	47 (4.6)	0.35 (0.19-0.61)	< 0.001	0.34 (0.19-0.62)	< 0.001
Additive			0.77 (0.65-0.92)	0.003	0.78 (0.65-0.92)	0.004

a Note: Logistic regression analyses adjusted for age, pre-pregnancy BMI, parity, abnormal pregnancy history and family history of diabetes. Abbreviations: GDM, gestational diabetes mellitus; SNP, single nucleotide polymorphism.

Then, we evaluated the combined effects on GDM occurrence by adding the number of variant alleles of the three significant SNPs (rs10511544-C, rs10756026-A and rs10809070-G). The “0” allele means subjects with wide-type homozygotes of the three SNPs; “1-6” alleles means carrying one to six variant alleles of the three SNPs. The results showed that the GDM risk was significantly decreased with an increasing number of variant alleles of the three SNPs in a dose-dependent manner (*P*_trend_ = 0.008) (Table 4). When compared with the subjects with wide-type homozygotes of the three SNPs, subjects carrying four to six variant alleles had a 46% decrease in GDM risk (95% CI = 0.37-0.81) (Table 4). The combined effects of the three SNPs on GDM occurrence were also evaluated by stratifying by age, pre-pregnancy BMI, parity, abnormal pregnancy history and family history of diabetes. No obvious evidence of heterogeneity associations for the combined effects of the three SNPs on GDM risk was observed (Supplementary Table S1). We also conducted stratified analyses on rs10511544, rs10756026 and rs10809070 with GDM susceptibility, respectively (Supplementary Table S2–S4). There was also no heterogeneity between the similar association strengths of the subgroups (*P* > 0.05).

**Table 4:** Cumulative effects of variant alleles on GDM susceptibility

Variables	GDM cases (n = 964) N (%)	Controls (n = 1021) N (%)	OR (95%CI)	*P*	OR (95%CI)^a^	*P*^a^
Combined effects of rs10511544-C, rs10756026-A and rs10809070-G						
0	546 (57.72)	538 (53.21)	1.00		1.00	
1-3	356 (37.63)	393 (38.87)	0.89 (0.74-1.08)	0.232	0.90 (0.75-1.09)	0.290
4-6	44 (4.65)	80 (7.91)	0.54 (0.37-0.80)	0.002	0.54 (0.37-0.81)	0.002
Trend			*P*^b^ = 0.005		*P*^b^ = 0.008	

a Note: Logistic regression analyses adjusted for age, pre-pregnancy BMI, parity, abnormal pregnancy history and family history of diabetes.

b *P* value of Cochran-Armitage's trend test. Abbreviation: GDM, gestational diabetes mellitus.

The linkage disequilibrium (LD) information of the three SNPs, calculated from genotyping data of the control group, is shown in Supplementary Table S5. We conducted haplotype analyses to evaluate the effect of the haplotype containing variant alleles of the three SNPs on GDM occurrence (Table 5). When compared with the most frequent ATC haplotype, the haplotype containing variant alleles of the three SNPs (CAG) was significantly associated with a decreased risk of GDM [adjusted (95% CI) = 0.77 (0.64-0.92), *P* = 0.005)], which was consistent with the single SNP analysis.

**Table 5:** Results of haplotype association analyses

Haplotype	GDM N (%)	Controls N (%)	OR (95%CI)	*P*
ATC	1464 (75.9)	1455 (71.3)	1.00	
CAG	249 (12.9)	329 (16.1)	0.77 (0.64-0.92)	0.005
CAC	148 (7.7)	163 (8.0)	0.95 (0.75-1.20)	0.661
Others^a^	67 (3.5)	95 (4.7)	0.69 (0.50-0.96)	0.025

Note: Logistic regression analyses adjusted for age, pre-pregnancy BMI, parity, abnormal pregnancy history and family history of diabetes. SNPs order: rs16847024, rs17429130 and rs4917356.

a Haplotypes with a frequency less than 5% in all three groups were combined as others. Abbreviation: GDM, gestational diabetes mellitus.

## DISCUSSION

As far as we know, this is the first study that has provided evidence that common SNPs in *PTPRD* might be associated with GDM susceptibility. We found that *PTPRD* rs10511544 A>C, rs10756026 T>A and rs10809070 C>G were significantly associated with a decreased risk of GDM. Additionally, GDM risk significantly decreased with an increasing number of variant alleles of the three SNPs in a dose-dependent manner.

*PTPRD* belongs to the R2A subfamily of protein tyrosine phosphatases (PTPs), which have been implicated in cancer, neural development, and diabetes [13]. *PTPRD* is expressed widely, including the brain, the pancreas and skeletal muscle. Multiple mRNA isoforms are expressed by alternative transcription start sites (TSSs) and/or alternative splicing in a tissue- and developmental-specific manner [14]. *PTPRD* is one of the most frequently inactivated genes across human cancers [15]. In gliomas, loss of PTPRD could cause aberrant activation of STAT3 and is closely associated with glioma progression [16]. *PTPRD*-deficient mice exhibit neonatal mortality, impaired learning and memory, posture and motor defects, and early growth retardation [17]. The R2A PTP subfamily includes *PTPRD*, *PTPRS* and *LAR*. These members are structurally very similar [14]. The overexpression of LAR in mouse skeletal muscle could cause whole-body insulin resistance [18]. The *PTPRS*- and *LAR*-deficient mice were demonstrated to have altered insulin sensitivity and glucose homeostasis [19]. Therefore, PTPRD may also play a role in the pathogenesis of T2D and GDM, and should be further characterized.

The SNPs rs10511544 A>C, rs10756026 T>A and rs10809070 C>G were located in the intron of *PTPRD*. Interestingly, the variant alleles of the three SNPs were associated with higher expression of *PTPRD*, according to the GTEx Portal (http://www.gtexportal.org) database, and PTPRD levels were reported to be lower in T2D patients [12], supporting the protective effect of the variant alleles of the three SNPs on GDM occurrence. Although this evidence for the three SNPs seems to be biologically plausible, lack of evidence for the functionality that links the three SNPs and *PTPRD* and then links the latter to GDM occurrence was one of the drawbacks of this study. Therefore, further functional analysis of the regions including the three SNPs is needed.

Our study had a number of strengths. First, our GDM cases and controls came from a systematic screening of pregnancy complications in a population-based, large study performed in Nanjing, and the two groups were well matched based on age and pre-pregnancy BMI, which may have reduced a potential selection bias. Moreover, the relatively large sample size in this study provided enough statistical power. However, some reported risk factors for GDM, such as poor diet, low physical activity, and polycystic ovarian syndrome [20, 21], were not considered for adjustment in this study. Our study was underpowered to detect associations of some of the SNPs with GDM, especially for the two SNPs reported in the T2D GWAS (rs17584499 and rs649891), probably because of their lower frequencies, which may have resulted in some associations being overlooked. The heterogeneity among populations and diseases (T2D and GDM) may also contribute to the negative results. Therefore, the results should be treated with caution, and validation is warranted.

Altogether, our study suggested that *PTPRD* loci are candidate susceptibility regions that have some marker SNPs for GDM in Han Chinese. Further studies performed in diverse populations with functional assays are needed to validate and extend our findings.

## MATERIALS AND METHODS

### Study subjects

The study was approved by the institutional review board of Nanjing Maternity and Child Health Care Institute (Nanjing, China), and the methods were carried out in accordance with the approved guidelines. The case-control study was conducted on the basis of a study population of more than 80,000 women who had pregnancy complications screening between March 2012 and February 2015 at Nanjing Maternity and Child Health Care Hospital (Nanjing, China). The GDM cases and controls were randomly selected from the maternal screening population using a computerized random number function. All participants were offered a glucose challenge test (GCT) between weeks 24 and 28 of gestation. The GDM cases were defined as pregnant women with fasting glucose concentration ≥ 5.5 mmol/L or 2-h plasma glucose concentration ≥ 8.0 mmol/L [22]. Women diagnosed with diabetes before pregnancy were excluded from this study. Pregnant women without diabetes were included as controls. The selected controls were matched to the GDM patients based on age and pre-pregnancy BMI and declared to have no previous metabolic diseases. As a result, 964 GDM cases and 1,021 controls consented to participate in the study. After written informed consent was obtained, each participant was scheduled for an interview using a structured questionnaire to collect demographic information and potential risk factors, such as age, pre-pregnancy height and weight, parity, abnormal pregnancy history and family history of diabetes. All cases and controls were unrelated ethnic Han Chinese.

### SNPs selection and genotyping

Two SNPs in *PTPRD* (rs17584499 and rs649891) reported in T2D GWAS and common *PTPRD* expression quantitative trait loci (eQTLs) SNPs were included for analysis. The selected eQTLs SNPs had minor allele frequency (MAF) larger than 0.05 in Han Chinese and were associated with *PTPRD* expression according to the GTEx Portal Database (last search date: December 2015). If SNPs were in high linkage disequilibrium (r^2^ > 0.8), we genotyped only one SNP. As a result, the two GWAS-reported SNPs in *PTPRD* (rs17584499 and rs649891) and six *PTPRD* eQTLs SNPs (rs10511544, rs10756026, rs10809070, rs12345848, rs1323500 and rs628731) were selected for genotyping (Table 2).

Genomic DNA was extracted from leukocyte pellets by traditional proteinase K digestion, followed by phenol-chloroform extraction and ethanol precipitation. All SNPs were genotyped using the Sequenom MassARRAY iPLEX platform (Sequenom Inc., CA). The information regarding the primers is shown in Supplementary Table S6. The genotyping assays were performed without knowledge of the subjects' case and control status. Two blank controls (water) in each 384-well plate were used for quality control, and more than 10% of the samples were randomly selected to repeat, yielding a 100% concordance. The success rates of genotyping for these SNPs were all above 98.5%.

### Statistical analyses

Differences of selected characteristics and genotype frequencies of SNPs between the GDM cases and controls were calculated using Student's *t*-test (for continuous variables) and χ^2^ test (for categorical variables). Both univariate and multivariate logistic regression analyses were used to estimate the associations between the genotypes and GDM risk by computing odds ratios (OR) and their 95% confidence intervals (CIs). The Cochran-Armitage test was used for trend analyses. The χ^2^-based Q test was used to assess the heterogeneity of associations among subgroups. Haploview was employed to analyse LD parameters (r^2^ and D'). PHASE software (v2.1) was used to estimate the haplotype frequencies based on the observed genotypes. All the statistical analyses were performed with SAS 9.1.3 software (SAS Institute, Cary, NC), and *P* < 0.05 in a two-sided test was considered statistically significant.

## SUPPLEMENTARY TABLES


