# Supplementary material for: Genetic variants in PTPRD and risk of gestational diabetes mellitus
Source: Oncotarget. 2016 Oct 12;7(46):76101–7. doi: 10.18632/oncotarget.12599 (PMC5340179; doi:10.18632/oncotarget.12599)
Supplement: Supplementary file 1 [file oncotarget-07-76101-s001.pdf]

Genetic variants in *PTPRD* and risk of gestational diabetes mellitus

SUPPLEMENTARY TABLES

Supplementary Table S1: Stratified analyses on the combined effects of the three SNPs with GDM susceptibility

Variables	0/1–2/3-6 allele (s) GDM cases (n = 964) N (%)	0/1–2/3-6 allele (s) Controls (n = 1021) N (%)	OR (95%CI)	*P*[b]	OR (95%CI)[a]	*P*[b]
Age, year						
<= 30	276/182/17	332/241/46	0.79 (0.65-0.97)	0.805	0.84 (0.68-1.03)	0.814
> 30	270/174/27	206/152/34	0.82 (0.66-1.02)		0.81 (0.65-1.01)	
Pre-pregnancy BMI, kg/m^2						
<= 22	277/190/23	282/195/40	0.87 (0.71-1.07)	0.323	0.87 (0.71-1.07)	0.416
> 22	269/166/21	256/198/40	0.75 (0.61-0.93)		0.77 (0.63-0.96)	
Parity						
Nulliparae	466/302/42	502/367/74	0.83 (0.71-0.97)	0.421	0.84 (0.72-0.98)	0.534
Multiparae	80/54/2	36/26/6	0.67 (0.41-1.11)		0.70 (0.40-1.21)	
Abnormal pregnancy history						
No	479/315/37	514/381/76	0.80 (0.69-0.94)	0.571	0.81 (0.70-0.95)	0.324
Yes	67/41/7	24/12/4	0.95 (0.53-1.67)		1.13 (0.59-2.14)	
Family history of diabetes						
No	440/298/39	466/330/70	0.86 (0.73-1.00)	0.108	0.87 (0.74-1.03)	0.084
Yes	106/58/5	72/63/10	0.61 (0.41-0.89)		0.60 (0.41-0.89)	

Note: [a] Logistic regression analyses adjusted for age, pre-pregnancy BMI, parity, abnormal pregnancy history and family history of diabetes (excluded the stratified factor in each stratum). [b] *P* -value for the heterogeneity test. Abbreviations: GDM, gestational diabetes mellitus; SNP, single nucleotide polymorphism; BMI, body mass index.

Supplementary Table S2: Stratified analyses on rs10511544 with GDM susceptibility

Variables	AA/CA/CC *(N)*		OR (95%CI)	*P*[b]	OR (95%CI)[a]	*P*[b]
	GDM cases (n = 964) N (%)	Controls (n = 1021) N (%)				
Age, year						
<= 30	283/182/18	339/236/50	0.79 (0.65-0.96)	0.509	0.84 (0.69-1.03)	0.875
> 30	279/166/30	220/142/34	0.87 (0.71-1.08)		0.86 (0.69-1.06)	
Pre-pregnancy BMI, kg/m^2						
<= 22	287/185/25	294/187/43	0.88 (0.72-1.08)	0.419	0.87 (0.71-1.07)	0.576
> 22	275/163/23	265/191/41	0.78 (0.63-0.96)		0.80 (0.65-0.99)	
Parity						
Nulliparae	478/299/ 44	520/355/ 78	0.85 (0.73-0.98)	0.669	0.85 (0.73-0.99)	0.867
Multiparae	84/49/4	39/23/6	0.76 (0.46-1.23)		0.81 (0.47-1.39)	
Abnormal pregnancy history						
No	495/308/ 39	534/367/ 80	0.81 (0.70-0.94)	0.361	0.82 (0.71-0.96)	0.244
Yes	67/40/9	25/11/4	1.06 (0.61-1.86)		1.21 (0.64-2.29)	
Family history of diabetes						
No	454/291/42	485/317/74	0.87 (0.74-1.02)	0.144	0.88 (0.75-1.04)	0.141
Yes	108/57/ 6	74/61/10	0.64 (0.44-0.94)		0.64 (0.43-0.94)	

Note: [a] Logistic regression analyses adjusted for age, pre-pregnancy BMI, parity, abnormal pregnancy history and family history of diabetes (excluded the stratified factor in each stratum). [b] *P* -value for the heterogeneity test. Abbreviations: GDM, gestational diabetes mellitus; SNP, single nucleotide polymorphism; BMI, body mass index.

Supplementary Table S3: Stratified analyses on rs10756026 with GDM susceptibility

Variables	TT/AT/AA *(N)*		OR (95%CI)	*P*[b]	OR (95%CI)[a]	*P*[b]
	GDM cases (n = 964) N (%)	Controls (n = 1021) N (%)				
Age, year						
<= 30	297/171/17	351/230/39	0.81 (0.66-0.99)	0.935	0.84 (0.68-1.04)	0.695
> 30	294/161/23	222/136/34	0.80 (0.64-0.99)		0.79 (0.63-0.98)	
Pre-pregnancy BMI, kg/m^2						
<= 22	304/172/23	298/184/35	0.86 (0.70-1.06)	0.365	0.85 (0.69-1.05)	0.517
> 22	287/160/17	275/182/38	0.75 (0.61-0.93)		0.77 (0.62-0.95)	
Parity						
Nulliparae	506/283/37	536/340/68	0.82 (0.70-0.96)	0.486	0.82 (0.70-0.96)	0.658
Multiparae	85/49/3	37/26/5	0.68 (0.41-1.12)		0.72 (0.41-1.24)	
Abnormal pregnancy history						
No	518/295/34	549/354/69	0.81 (0.69-0.94)	0.968	0.81 (0.69-0.95)	0.553
Yes	73/37/6	24/12/4	0.82 (0.46-1.45)		0.99 (0.52-1.88)	
Family history of diabetes						
No	478/278/34	499/303/65	0.85 (0.72-0.99)	0.115	0.86 (0.73-1.02)	0.115
Yes	113/54/6	74/63/8	0.61 (0.42-0.90)		0.61 (0.41-0.90)	

Note: [a] Logistic regression analyses adjusted for age, pre-pregnancy BMI, parity, abnormal pregnancy history and family history of diabetes (excluded the stratified factor in each stratum). [b] *P* -value for the heterogeneity test. Abbreviations: GDM, gestational diabetes mellitus; SNP, single nucleotide polymorphism; BMI, body mass index.

Supplementary Table S4: Stratified analyses on rs10809070 with GDM susceptibility

Variables	CC/GC/GG (*N*)		OR (95%CI)	*P*[b]	OR (95%CI)[a]	*P*[b]
	GDM cases (n = 964) N (%)	Controls (n = 1021) N (%)				
Age, year						
<= 30	335/137/4	418/179/27	0.78 (0.62-0.98)	0.941	0.82 (0.65-1.04)	0.778
> 30	356/104/12	282/94/20	0.79 (0.62-1.02)		0.78 (0.60-1.00)	
Pre-pregnancy BMI, kg/m^2						
<= 22	351/132/8	359/139/26	0.80 (0.64-1.01)	0.649	0.81 (0.64-1.03)	0.720
> 22	340/109/8	341/134/21	0.74 (0.58-0.95)		0.76 (0.59-0.98)	
Parity						
Nulliparae	588/208/16	653/255/44	0.80 (0.67-0.95)	0.447	0.80 (0.67-0.96)	0.617
Multiparae	103/33/0	47/18/3	0.63 (0.35-1.14)		0.67 (0.34-1.30)	
Abnormal pregnancy history						
No	612/207/13	669/266/45	0.74 (0.62-0.89)	0.136	0.75 (0.63-0.90)	0.075
Yes	79/34/3	31/7/2	1.29 (0.64-2.64)		1.53 (0.71-3.28)	
Family history of diabetes						
No	566/200/13	601/233/41	0.78 (0.65-0.94)	0.823	0.80 (0.66-0.96)	0.790
Yes	125/41/3	99/40/6	0.74 (0.49-1.14)		0.75 (0.48-1.15)	

Note: [a] Logistic regression analyses adjusted for age, pre-pregnancy BMI, parity, abnormal pregnancy history and family history of diabetes (excluded the stratified factor in each stratum). [b] *P* -value for the heterogeneity test. Abbreviations: GDM, gestational diabetes mellitus; SNP, single nucleotide polymorphism; BMI, body mass index.

Supplementary Table S5: Linkage disequilibrium (LD) information of the three SNPs

SNP	rs10511544	rs10756026	rs10809070
rs10511544	--	0.907[a]	0.943[a]
rs10756026	0.782[b]	--	0.869[a]
rs10809070	0.535[b]	0.483[b]	--

[a] D'; [b] R^2

Supplementary Table S6 Information of primers for Sequenom MassARRAY iPLEX assays

SNP_ID	2nd-PCR Primer	1st-PCR Primer	Extend Primer
rs17584499	ACGTTGGATGCTTCCT TTAAACAGTACACC	ACGTTGGATGTGGGCCCA AGAAAAGACAAC	AAACAGTACACCTTTCTGTATT
rs649891	ACGTTGGATGAAATTAG GACATTCAACAC	ACGTTGGATGGAGGTATT TGTTGTTGTATA	GATTTAGTCTCTTATA TTTAAGATAGTC
rs10511544	ACGTTGGATGGGTTGG CACTGTTTGAACTG	ACGTTGGATGGGGTTTAGCT AAAATGCTTGG	TGATGATAATATGAA TTAACTTATGG
rs10756026	ACGTTGGATGGCATTT TCTTAACACTTTCC	ACGTTGGATGGTTTGCGT TTTTCCCAACAAG	AACACTTTCCTAAT ATAAAATTTTTTT
rs10809070	ACGTTGGATGCATTTCT TGTATGTACTTTC	ACGTTGGATGCTTTCGGT ATTGATTTGTCC	GGTAAGCCTTAGT AACTGATTAA
rs12345848	ACGTTGGATGTGCTAATT GTGACGCTACCC	ACGTTGGATGGATGGTTT CTATAGTTTAGAC	TTGTCAATTTTTACCTTTGGT
rs1323500	ACGTTGGATGCCCAAATA AAAGCATTTTCC	ACGTTGGATGCTCAAGCA TAGTAGAAAAACC	GCATTTTCCAACTTTGATAATTTT
rs628731	ACGTTGGATGCCTGAGTT CTAACTTGATTG	ACGTTGGATGAAACACTCC TCAGGGAATGC	AGAGACTGTTTGTTATGATTGC
